# Epidemiology of bone cancer in Saudi Arabia: a nationwide population-based study (2004–2020)

**DOI:** 10.3389/fonc.2026.1780642

**Published:** 2026-06-26

**Authors:** Ibrahim G. Alghamdi, Hasan A Alaidarous, Murad I Alghamdi, Khalid A Alghamdi, Musharraf A Alghamdi, Mazen S Alghamdi, Anas A Alzahrani, Tariq A Alghamdi, Osama M Alomari, Khalid S Alghamdi, Mohammed M Alzahrani, Ragad A Alzahrani, Hazim H Alsadi

**Affiliations:** 1 Public Health Department, College of Applied Medical Sciences, University of Al-Baha, Al-Baha, Saudi Arabia; 2 Surgery Department, College of Medicine, University of Al-Baha, Al-Baha, Saudi Arabia; 3 College of Medicine, University of Al-Baha, Al-Baha, Saudi Arabia; 4 General Directorate of Health Affairs Al-Baha, Ministry of Health, Al-Baha, Saudi Arabia

**Keywords:** age-standardized incidence rate, bone cancer, crude incidence rate, epidemiology, Saudi Arabia, Saudi cancer registry, temporal patterns

## Abstract

**Background:**

Bone cancer is a rare malignancy worldwide, with incidence patterns that vary by age, sex, and geographic region. In Saudi Arabia, however, comprehensive population-based evidence describing the national epidemiology of bone cancer remains limited. This study aimed to describe bone cancer incidence in Saudi Arabia according to age group, sex, calendar year, and administrative region, with particular emphasis on age-standardized incidence rates (ASIRs).

**Methods:**

A retrospective population-based descriptive study was conducted using data from the Saudi Cancer Registry. All primary bone cancer cases diagnosed between 2004 and 2020 were included. Incidence patterns were summarized using frequencies, age-specific incidence rates, crude incidence rates (CIRs), and ASIRs, stratified by sex, age group, year of diagnosis, and region. Statistical analyses were performed using SPSS software version 30.

**Results:**

Between 2004 and 2020, a total of 2,275 primary bone cancer cases were recorded in Saudi Arabia, including 1,318 males (57.9%) and 957 females (42.1%). Bone cancer accounted for approximately 2.0% of all cancers among males and 0.9% among females. Mean ASIRs were higher in males (≈1.0 per 100,000) than females (≈0.7 per 100,000), while CIRs remained below 2.0 per 100,000 throughout the study period. Age-specific incidence showed a clear adolescent peak, most prominent in the 15–19-year age group, followed by the 10–14-year group. Regional variation in ASIRs was observed, with higher rates in Al-Jouf and Najran and persistently lower rates in Jazan.

**Conclusion:**

Bone cancer in Saudi Arabia is rare but demonstrates distinct variation by sex, age, and region. The observed male predominance and adolescent peak are consistent with international epidemiological patterns. Continued enhancement of population-based cancer surveillance is essential to support accurate epidemiological assessment and informed public health planning.

## 1 Introduction

Primary bone cancers are rare malignant tumours arising from bone and articular cartilage, comprising a heterogeneous group dominated by osteosarcoma, chondrosarcoma, and Ewing sarcoma. Despite their rarity, these tumours carry disproportionate clinical impact because they frequently occur at biologically vulnerable life stages (rapid skeletal growth and later-life comorbidity), often require complex multimodal treatment, and can lead to substantial disability and premature mortality (1).

Globally, population-based evidence confirms that bone cancer incidence and mortality are low in absolute terms but highly heterogeneous by sex, geography, and health-system capacity. Using Global Burden of Disease Study (GBD) 2021 and the Cancer Incidence in Five Continents (CI5) resources, recent international analyses show consistently higher age-standardised incidence in males than females, with wide cross-registry variation (male ASRs reaching 4 per 100,000 in some settings, while approaching zero in others), alongside marked regional contrasts in mortality trajectories over time declining in many high-income settings but persisting or rising in several middle-resource contexts. These global patterns support the epidemiologic expectation that observed differences may reflect a mixture of true risk variation, diagnostic access, and registry completeness rather than a single uniform worldwide trend (1).

Within the Middle East, robust national epidemiologic descriptions have historically been less common than in North America and Western Europe, and published registry-based work often focuses on major subtypes such as osteosarcoma. A national population-based study from Iran (2008–2014) reported an osteosarcoma ASIR of 3.02 per million person-years (equivalent to approximately 0.30 per 100,000; note that the Iranian study used per-million denominators whereas the present study reports per 100,000), a male-to-female ratio of 1.54:1, and a peak frequency at 15–19 years, illustrating the same adolescent concentration and male excess that is repeatedly described internationally, while also highlighting the value of high-quality registry infrastructure for interpreting regional patterns (2).

In Saudi Arabia, although the Saudi Cancer Registry provides national cancer surveillance, detailed long-term descriptions of primary bone cancer stratified by sex, age group, calendar year, and administrative region remain limited. Generating such population-based evidence is essential to establish a national epidemiological baseline, enable fair geographic comparison using age-standardised measures, and support future analytic studies exploring the determinants of observed variation. Therefore, the present study uses Saudi Cancer Registry data (2004–2020) to describe the epidemiology of primary bone cancer in Saudi Arabia by sex, age, time, and region using age-specific, crude, and age-standardised incidence measures.

## 2 Methods

### 2.1 Study design and data source

This study employed a retrospective descriptive epidemiological design utilizing data obtained from the Saudi Cancer Registry (SCR) for the period January 2004 to December 2020. The SCR, established in 1994 by the Saudi Ministry of Health, is a population-based registry that systematically collects and compiles data on all newly diagnosed cancer cases across the Kingdom. The registry reports are publicly available and contain anonymized data; therefore, ethical approval was not required for this analysis. Data were extracted for all histologically confirmed primary malignant bone tumors, coded according to the International Classification of Diseases for Oncology, Third Edition (ICD-O-3). Specifically, cases were identified using topography codes C40.0–C41.9 (bones, joints, and articular cartilage). Cases with secondary or metastatic bone tumors, benign neoplasms, and tumors of uncertain behavior were excluded. The SCR dataset provides comprehensive information on cancer incidence, including the year of diagnosis, patient age, sex, and region of residence at the time of diagnosis.

### 2.2 Study population and case selection

All Saudi nationals diagnosed with primary malignant bone tumors during the 17-year study period were included. Cases were stratified by sex (male, female), age group, and geographic region corresponding to the 13 administrative regions of Saudi Arabia. The age groups were classified in five-year intervals (0–4, 5–9, 10–14, 15–19, 20–24, …, ≥75 years), consistent with the grouping approach adopted by the World Health Organization (WHO) for cancer surveillance.

### 2.3 Incidence measures

Crude incidence rates (CIRs), age-specific incidence rates, and age-standardized incidence rates (ASIRs) were obtained directly from published Saudi Cancer Registry reports, where these indicators are routinely calculated using standardized epidemiological methods. ASIRs were computed by the registry using the direct standardization method with the World Standard Population as the reference. No recalculation of incidence rates was performed in the present study.

### 2.4 Descriptive and visual analyses

Incidence patterns were summarized using descriptive statistics, including counts, proportions, means, medians, interquartile ranges, and ranges, as appropriate. Prior to conducting inferential analyses, the distribution of incidence rate data was assessed for normality using graphical methods and formal normality tests. Given the rarity of bone cancer and the presence of years with zero incidence in several regions and age groups, descriptive summaries were emphasized to characterize overall patterns.

To assess differences in incidence across age groups and geographic regions, inferential statistical analyses were performed separately for males and females using one-way analysis of variance (ANOVA), where assumptions of approximate normality were considered acceptable. Prior to conducting ANOVA, the homogeneity of variance assumption was assessed using Levene’s test, and the distribution of residuals was evaluated with the Shapiro-Wilk test. Given the violation of both assumptions in several analyses, Kruskal-Wallis non-parametric tests were performed as sensitivity analyses to confirm the robustness of parametric findings. Effect sizes were quantified using eta-squared (η²) where appropriate. When overall statistical significance was detected, Tukey’s honestly significant difference (HSD) test was applied for *post-hoc* pairwise comparisons. Each observation in the ANOVA represents an annual incidence rate for a given stratum (age group or region), yielding N = 272 observations (16 age groups × 17 years) for age-specific analyses and N = 221 observations (13 regions × 17 years) for regional analyses. Temporal descriptions of incidence patterns are based on visual inspection of annual rates and should be interpreted as observational; no formal trend analysis (e.g., joinpoint regression or Poisson time-series models) was performed.

In addition, regional and temporal variability was visually explored using heatmap visualizations to illustrate patterns in age-standardized incidence rates (ASIRs), crude incidence rates, and age-specific incidence rates across years and regions. Heatmaps were employed as descriptive visualization tools to support interpretation of spatial and temporal patterns and did not, on their own, imply statistical significance or monotonic temporal trends.

### 2.5 Ethical considerations

This study utilized publicly available, aggregated data from the Saudi Cancer Registry. All data were anonymized, and no individual-level identifiers were accessed. Ethical approval and informed consent were not required.

## 3 Results

### 3.1 Bone cancer in Saudi males

#### 3.1.1 Total cases and age distribution

**Figure 1:**
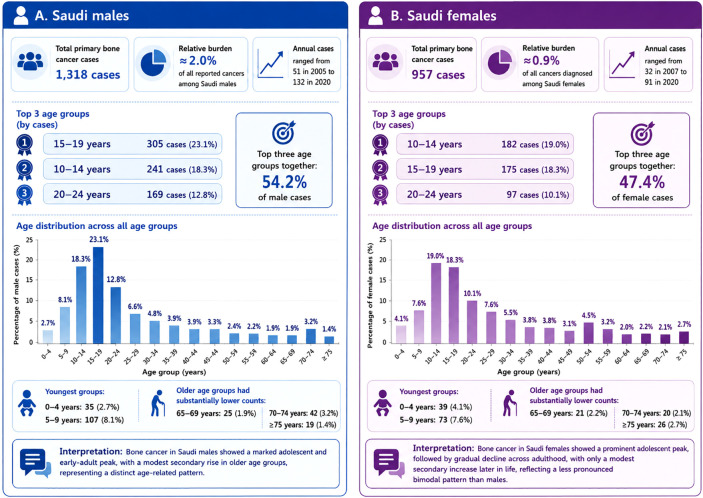
Age distribution of bone cancer cases in Saudi Arabia (2004–2020). **(A)** Saudi males and **(B)** Saudi females. The image illustrates the number and proportion of bone cancer cases by age group, demonstrating a prominent adolescent peak in both sexes, with a higher relative burden among males and a less pronounced secondary rise in older age groups.

- Between 2004 and 2020, a total of 1,318 primary bone cancer cases were reported among Saudi males. The annual number of cases ranged from 51 in 2005 to 132 in 2020, demonstrating an overall upward pattern in the recorded number of cases, although year-to-year fluctuations were observed and no formal trend test was applied. Overall, bone cancer constituted approximately 2.0% of all reported cancers among Saudi males during the study period. Age-group analysis revealed that the 15–19-year group accounted for the largest proportion, with 305 cases (23.1%), followed by 10–14 years with 241 cases (18.3%), and 20–24 years with 169 cases (12.8%). Collectively, these three age categories represented over half (54.2%) of all male bone cancer cases, underscoring the marked predominance of the disease among adolescents and young adults.
- In contrast, incidence was substantially lower in older age groups, including 65–69 years (25 cases, 1.9%), 70–74 years (42 cases, 3.2%), and ≥75 years (19 cases, 1.4%). The youngest groups (0–4 and 5–9 years) comprised 35 (2.7%) and 107 (8.1%) cases, respectively. **Table 1**, **Figure 1A** demonstrates this distinct age-related pattern, highlighting a major peak during adolescence and early adulthood, with a modest secondary rise observed in older age groups.

**Table 1:** Distribution of bone cancer cases and age-specific incidence rates by gender and age group, Saudi Arabia, 2004–2020.

Age group (years)	Males (N = 1,318)				Females (N = 957)			
	n	%	Mean IR	SD	n	%	Mean IR	SD
0–4	35	2.7	0.19	0.16	39	4.1	0.23	0.13
5–9	107	8.1	0.56	0.24	73	7.6	0.43	0.22
10–14	241	18.3	1.39	0.34	182	19.0	1.14	0.46
15–19	305	23.1	1.82	0.42	175	18.3	1.00	0.35
20–24	169	12.8	0.98	0.38	97	10.1	0.61	0.45
25–29	87	6.6	0.56	0.28	73	7.6	0.46	0.32
30–34	63	4.8	0.49	0.26	53	5.5	0.36	0.23
35–39	51	3.9	0.44	0.27	36	3.8	0.30	0.30
40–44	43	3.3	0.45	0.23	36	3.8	0.38	0.29
45–49	44	3.3	0.57	0.37	30	3.1	0.44	0.34
50–54	32	2.4	0.46	0.32	43	4.5	0.72	0.73
55–59	29	2.2	0.62	0.58	31	3.2	0.65	0.58
60–64	25	1.9	0.79	0.62	19	2.0	0.57	0.51
65–69	19	1.4	1.06	0.73	21	2.2	0.75	0.87
70–74	25	1.9	1.24	1.17	20	2.1	1.12	0.78
≥75	42	3.2	1.81	1.55	26	2.7	1.26	0.93
**Total**	**1,318**	**100**	**—**	**—**	**957**	**100**	**—**	**—**

IR , incidence rate per 100,000 population; SD , standard deviation. Rates were calculated from 17-year annual data (2004–2020). Bold values indicate total counts and percentages for each sex.

#### 3.1.2 Age-specific incidence rate in Saudi males

Age-specific incidence rates of bone cancer in Saudi males demonstrated a consistent bimodal age pattern (i.e., an adolescent peak in the 10–19 age range and a secondary rise among adults aged ≥60 years) over the study period (2004–2020). Incidence rates were lowest in early childhood (0–4 years: median 0.1, mean 0.2 per 100,000), followed by a gradual increase in school-aged children (5–9 years: median 0.6, mean 0.6 per 100,000). Rates increased further during early adolescence (10–14 years: median 1.4, mean 1.4 per 100,000) and reached a first peak in late adolescence (15–19 years: median 1.9, mean 1.8 per 100,000), representing the highest average incidence across all age groups.

**Figure 2:**
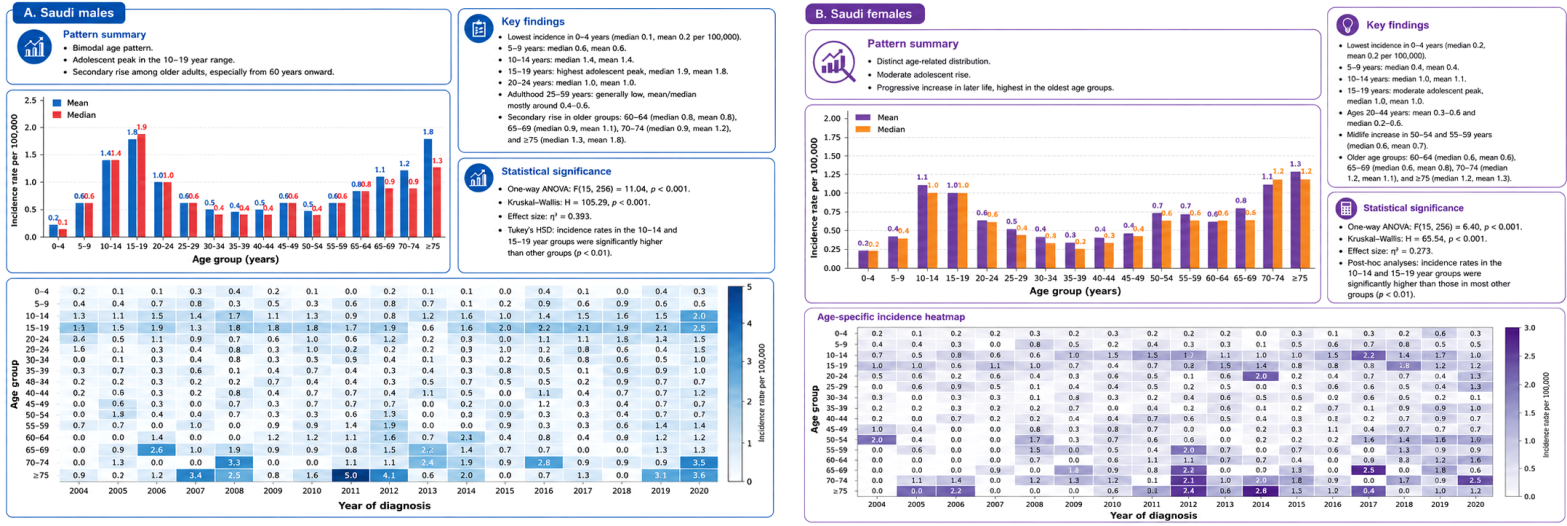
Age-specific incidence rates of bone cancer in Saudi Arabia (2004–2020). **(A)** Saudi males and **(B)** Saudi females. Age-specific incidence rates (per 100,000) are shown across age groups, revealing a characteristic bimodal distribution with a marked adolescent peak in males and a comparatively moderate adolescent peak followed by a progressive increase in older age groups among females.

After adolescence, incidence rates declined during young adulthood (20–24 years: median 1.0, mean 1.0 per 100,000) and remained relatively low throughout adulthood (25–59: range of mean and median (0.4 to 0.6 per 100,000). A secondary increase was observed in older age groups, with incidence rates rising from 60–64 years (median 0.8, mean 0.8) and 65–69 years (median 0.9, mean 1.1) to 70–74 years (median 0.9, mean 1.2), reaching the highest rates among the elderly aged ≥75 years (median 1.3, mean 1.8 per 100,000). These differences in age-specific incidence rates were statistically significant, as demonstrated by a one-way ANOVA showing a significant variation across age groups (F(15, 256) = 11.04, p < 0.001; Kruskal-Wallis H = 105.29, p < 0.001; η² = 0.393). *Post-hoc* analysis using Tukey’s HSD test revealed that age-specific incidence rates in the 10–14 and 15–19 age groups were significantly higher than other groups (p < 0.01), confirming a statistically meaningful elevation during adolescence (**Figure 2A**).

#### 3.1.3 Crude incidence rate in Saudi males

Across regions, the mean crude incidence rate (CIR) of bone cancer among Saudi males ranged from 0.4 to 1.2 per 100,000, while median values ranged from 0.3 to 1.0 per 100,000 over the study period. The lowest crude incidence levels were consistently observed in Jazan, whereas the highest mean and median CIRs were recorded in Al-Jouf and Najran, indicating notable regional heterogeneity in the population burden of bone cancer. These observed differences were statistically significant [F(12, 208) = 2.27, p = 0.010; Kruskal-Wallis H = 22.88, p = 0.029], with *post-hoc* analysis showing that Al-Jouf had significantly higher CIR than Jazan (p < 0.01), while no other regional differences reached statistical significance.

**Figure 3:**
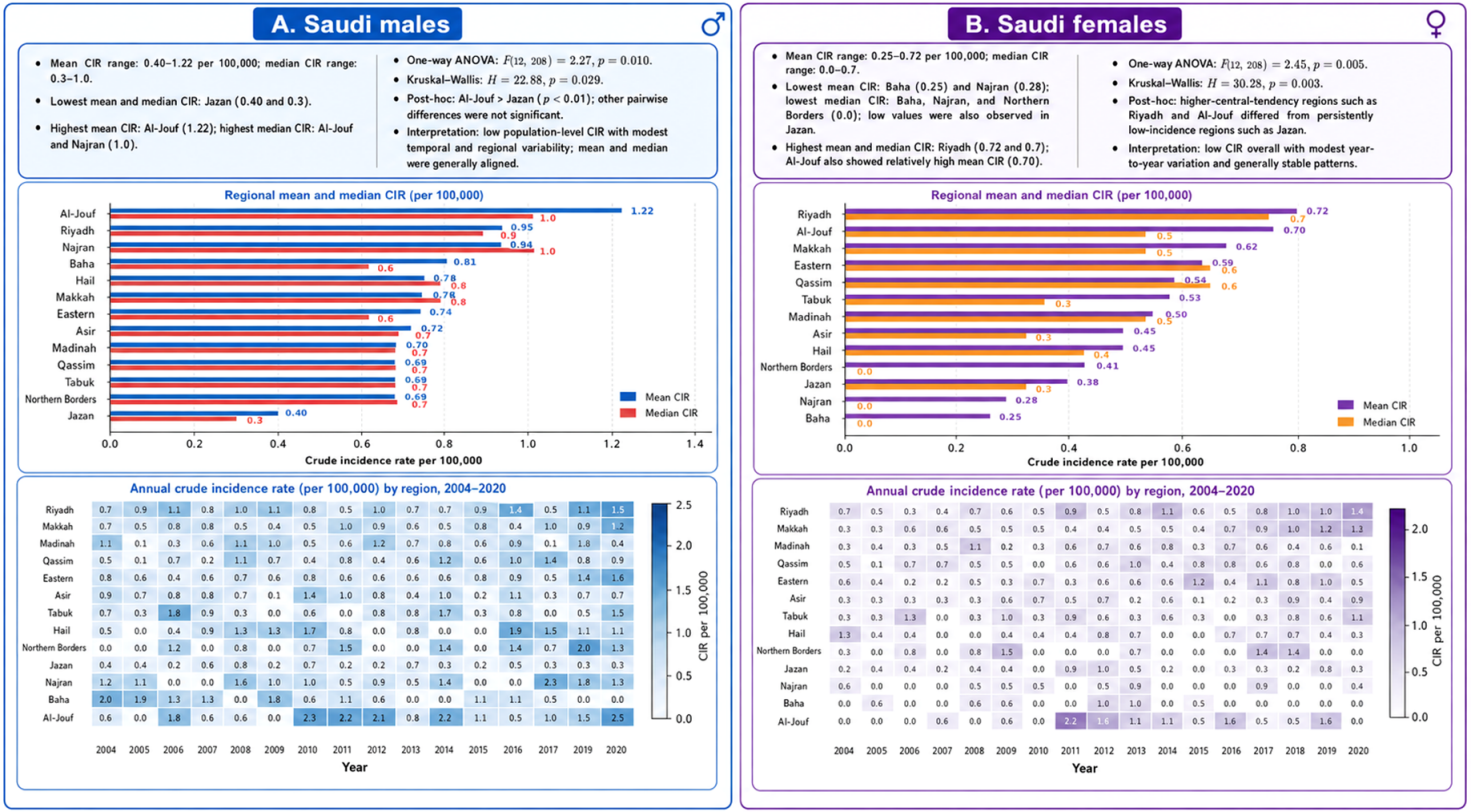
Regional distribution of crude incidence rates (CIRs) of bone cancer in Saudi Arabia (2004–2020). **(A)** Saudi males and **(B)** Saudi females. Mean and median crude incidence rates (per 100,000) are presented by administrative region, illustrating overall low incidence levels with measurable geographic variation and consistently lower rates in selected regions.

Overall, the crude incidence rate of bone cancer among Saudi males exhibited modest temporal and regional variation between 2004 and 2020. CIR values remained relatively low across most regions, reflecting the rarity of bone cancer at the population level. Across calendar years, the majority of regions demonstrated largely stable CIR patterns with minor year-to-year fluctuations rather than sustained monotonic trends. Mean and median CIR values were closely aligned in most regions, suggesting limited influence of extreme annual values and generally stable crude incidence patterns over time (**Figure 3A**).

#### 3.1.4 Age-standardised incidence rate in Saudi males

Age-standardized incidence rates (ASIRs) of bone cancer among Saudi males demonstrated clear regional heterogeneity over the study period (2004–2020). Mean ASIR values ranged from approximately 0.4 per 100,000 in Jazan to 1.3 per 100,000 in Al-Jouf, while median values varied between 0.3 and 1.0 per 100,000, reflecting generally low but geographically uneven incidence patterns. Regions such as Al-Jouf and Najran consistently exhibited higher ASIRs across multiple calendar years, whereas Jazan maintained persistently low rates throughout the study period. In most regions, mean and median ASIR values were closely aligned, suggesting relatively stable temporal patterns with limited influence of extreme annual fluctuations, despite occasional year-specific peaks in selected regions.

**Figure 4:**
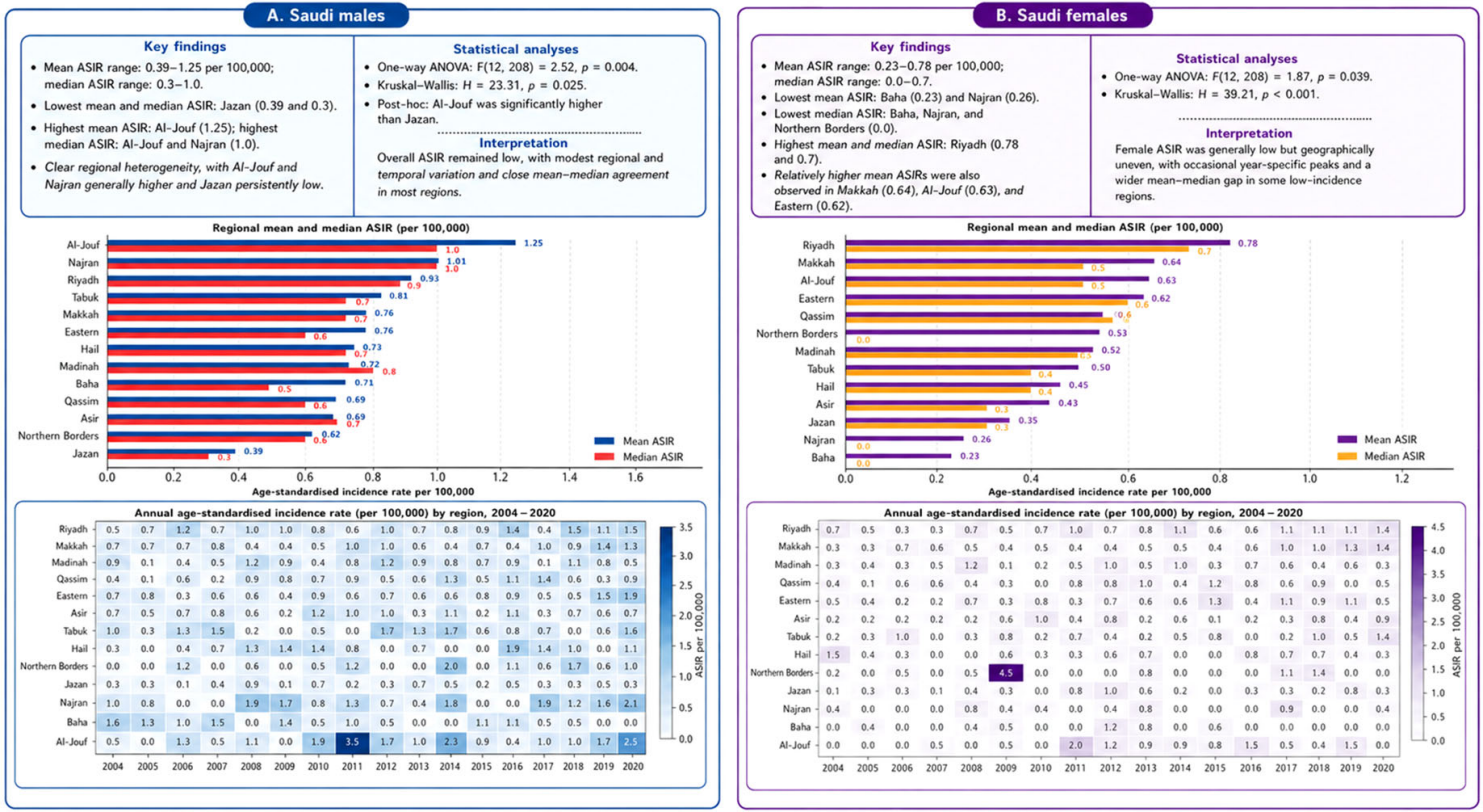
Age-standardized incidence rates (ASIRs) of bone cancer in Saudi Arabia by region (2004–2020). **(A)** Saudi males and **(B)** Saudi females. The figure displays mean and median ASIRs (per 100,000) across regions, highlighting significant geographic heterogeneity among males and lower but still variable incidence patterns among females.

Statistical analysis confirmed that these regional differences were unlikely to be due to random variation alone. One-way analysis of variance (ANOVA) revealed a statistically significant difference in ASIR across administrative regions [F(12, 208) = 2.52, p = 0.004; Kruskal-Wallis H = 23.31, p = 0.025 0.001]*. Post-hoc* comparisons using Tukey’s honestly significant difference (HSD) test identified significantly higher ASIRs in Al-Jouf compared with Jazan. However, no other pairwise regional comparisons reached statistical significance (**Figure 4A**).

### 3.2 Bone cancer in Saudi females

#### 3.2.1 Total cases and age distribution

From 2004 to 2020, a total of 957 primary bone cancer cases were documented among Saudi females, with annual case counts fluctuating between 32 cases in 2007 and 91 cases in 2020. Unlike the more pronounced upward trajectory observed in males, female case numbers demonstrated a more variable pattern over time, although a general increase was evident during the latter years of the study period. Overall, bone cancer represented approximately 0.9% of all cancers diagnosed among Saudi females, indicating a lower relative burden compared with males.

Age-specific analysis revealed a concentration of cases within younger age groups, particularly during adolescence. The 10–14-year age group constituted the largest share, accounting for 182 cases (19.0%), followed closely by 15–19 years with 175 cases (18.3%), and 20–24 years with 97 cases (10.1%). Together, these age categories comprised 47.4% of all female bone cancer cases, highlighting a strong predilection for occurrence during early life stages. In contrast, substantially fewer cases were observed among older adults, including 65–69 years (21 cases, 2.2%), 70–74 years (20 cases, 2.1%), and ≥75 years (26 cases, 2.7%). The youngest age groups, 0–4 years and 5–9 years, accounted for 39 (4.1%) and 73 (7.6%) cases, respectively.

As illustrated in **Table 1**, **Figure 1B**, the age distribution among females demonstrates a prominent adolescent peak, followed by a gradual decline across adulthood, with only a modest secondary increase in later life—reflecting a distinct but less pronounced bimodal pattern compared with males (**Table 1**; **Figure 1B**).

#### 3.2.2 Age-specific incidence rate in Saudi females

Age-specific incidence rates of bone cancer among Saudi females revealed a distinct age-related distribution over the study period (2004–2020), characterized by early-life stability, a moderate adolescent rise, and a pronounced increase in older age groups. Incidence rates were minimal in early childhood (0–4 years: median 0.2, mean 0.2 per 100,000) and remained low during late childhood (5–9 years: median 0.4, mean 0.4 per 100,000). A gradual elevation was observed during early adolescence (10–14 years: median 1.0, mean 1.1 per 100,000), followed by a modest peak in late adolescence (15–19 years: median 1.0, mean 1.0 per 100,000), which was less pronounced than that observed among males (**Figure 2B**).

In contrast to males, incidence rates among females declined more sharply during young and middle adulthood, remaining relatively low across ages 20–44 years (mean and median range: 0.3–0.6 per 100,000). From midlife onward, a progressive increase became evident, with higher incidence observed in the 50–54 (median 0.6, mean 0.7 per 100,000) and 55–59 age groups (median 0.6, mean 0.7 per 100,000). The most marked elevation occurred in older females, with rates increasing across 60–64 (median 0.6, mean 0.6), 65–69 (median 0.6, mean 0.8), and 70–74 years (median 1.1, mean 1.2 per 100,000), reaching the highest levels among those aged ≥75 years (median 1.2, mean 1.3 per 100,000). Overall differences in age-specific incidence rates across female age groups were statistically significant (F(15, 256) = 6.40, p < 0.001; Kruskal-Wallis H = 65.54, p < 0.001; η² = 0.273), with post-hoc analyses indicating that the incidence rates among adolescents (10–14 and 15–19 years) were significantly higher than those observed in most other age groups (p < 0.01), supporting the presence of a prominent adolescent peak in female bone cancer incidence.

#### 3.2.3 Crude incidence rate in Saudi females

Across administrative regions, the crude incidence rate (CIR) of bone cancer among Saudi females demonstrated measurable geographic variation over the study period. Mean CIR values ranged from approximately 0.3 to 0.7 per 100,000, while median values varied between 0.3 and 1.0 per 100,000, reflecting generally low population-level incidence. The lowest crude incidence levels were consistently observed in Jazan, whereas comparatively higher mean and median CIRs were noted in Riyadh and Al-Jouf, indicating discernible regional heterogeneity in female bone cancer burden. Statistical evaluation confirmed that these regional differences were unlikely to be attributable to random variation alone. One-way analysis of variance (ANOVA) demonstrated a statistically significant difference in CIR across regions [F(12, 208) = 2.45, p = 0.005; Kruskal-Wallis H = 30.28, p = 0.003]. *Post-hoc* comparisons indicated that regions with higher central tendency measures, particularly Al-Jouf and Riyadh, differed significantly from consistently low-incidence regions such as Jazan, while no widespread pairwise differences were observed among the remaining regions.

Overall, crude incidence rates among Saudi females remained low and relatively stable over time, with most regions exhibiting modest year-to-year fluctuations rather than sustained increasing or decreasing trends. The close agreement between mean and median CIR values across regions suggests limited influence of extreme annual values, reinforcing the interpretation of a generally stable crude incidence pattern. Nevertheless, as crude rates do not account for regional differences in age structure, these findings underscore the importance of age-standardized measures for more precise geographic comparisons (**Figure 3B**).

#### 3.2.4 Age-standardised incidence rate in Saudi females

Age-standardized incidence rates (ASIRs) of bone cancer among Saudi females during the period 2004–2020 were consistently low across all administrative regions, with limited but measurable geographic variation. Mean ASIR values generally ranged from approximately 0.2 to 0.8 per 100,000 females, while median values were frequently close to zero in several regions, reflecting the rarity of bone cancer and the occurrence of multiple calendar years with no reported cases. Relatively higher average ASIRs were observed in regions such as Riyadh, and Makkah, whereas persistently lower rates characterized regions including Jazan, Najran, and Baha. Temporal assessment demonstrated irregular year-to-year fluctuations rather than sustained monotonic trends, with occasional isolated peaks in specific regions and years. Statistical evaluation identified a modest significant difference in ASIR across regions (F(12, 208) = 1.87, p = 0.039); Kruskal-Wallis H = 39.21, p < 0.001), suggesting that observed regional variation is unlikely to be entirely attributable to random variation alone (**Figure 4B**).

## 4 Discussion

### 4.1 Overall burden of bone cancer and sex differences

This population-based analysis provides one of the most comprehensive national descriptions of bone cancer epidemiology in Saudi Arabia, revealing a consistently higher burden among males compared with females across the study period. Males accounted for approximately 58% of all reported cases, and bone cancer represented a larger proportion of total malignancies among males (2.0%) than among females (0.9%), indicating a clear sex disparity in disease occurrence.

This male predominance aligns closely with international observations. Global cancer surveillance data have consistently reported higher incidence rates of primary bone tumors in males, a pattern attributed to a combination of biological susceptibility, differences in skeletal growth dynamics, and potential sex-related variations in exposure to environmental or occupational risk factors (3, 4).

Importantly, the sex difference observed in the present study was not limited to crude case counts but was also evident in age-specific and age-standardized incidence measures. This consistency across epidemiological metrics suggests that the observed disparity reflects a genuine biological and demographic phenomenon rather than an artifact of population structure or reporting practices (5, 6).

### 4.2 Age-related patterns and the adolescent peak

A prominent age-related pattern was observed in the incidence of bone cancer among both Saudi males and females, characterized by a marked concentration of cases during adolescence and early adulthood. In the present study, more than half of male cases and nearly half of female cases occurred between the ages of 10 and 24 years, with a clear peak during late adolescence. This age distribution is a well-recognized epidemiological hallmark of primary malignant bone tumors and has been consistently reported across diverse populations worldwide (3, 7).

Age-specific incidence rates further substantiated this pattern, demonstrating a sharp rise in incidence during puberty, reaching maximal levels in the 15–19-year age group, followed by a decline in early adulthood. Similar adolescent peaks have been documented in population-based studies and international cancer registries, where bone cancer incidence increases rapidly during the second decade of life (4, 8).

The biological plausibility of this adolescent peak has been extensively discussed in the literature. Rapid longitudinal bone growth, increased osteoblastic activity, and heightened cellular proliferation at the growth plates during puberty are thought to create a biologically vulnerable window for malignant transformation (5, 6). Hormonal changes, particularly involving growth hormone and insulin-like growth factor-1 (IGF-1), have also been implicated in promoting tumorigenesis during this developmental period (9, 10). It should be noted that these mechanistic pathways are derived from experimental and clinical literature; the present registry-based study did not directly assess hormonal levels, growth factor expression, or cellular proliferation markers.

Although both sexes exhibited an adolescent peak, the magnitude of this increase was consistently greater among males than females, as reflected in higher age-specific and age-standardized incidence rates. This sex differential during adolescence has been observed globally and may reflect sex-specific differences in growth velocity, peak bone mass acquisition, and endocrine regulation (3, 4).

### 4.3 Late-onset increase and sex differences in older ages

In the present study, a secondary increase in bone cancer incidence was observed in older age groups, particularly among individuals aged ≥60 years, following the prominent adolescent peak. This pattern was evident in both sexes but was more pronounced among Saudi males, as reflected by rising age-specific incidence rates in the ≥70-year age categories and higher mean and median values among males compared with females. These findings confirm a bimodal age distribution of bone cancer within the Saudi population, consistent with patterns reported in population-based studies from other regions (11, 12).

Among Saudi males, age-specific incidence rates increased progressively from the 60–64-year group onward, reaching their highest levels in those aged ≥75 years. A similar but less pronounced late-onset rise was observed among Saudi females, where incidence remained relatively low during middle adulthood and increased gradually in older age groups. Comparable sex-specific differences in late-life incidence have been reported previously, with older males often demonstrating higher incidence and poorer outcomes than females (13, 14).

The late-onset increase observed in this study may reflect age-related biological mechanisms, including cumulative genomic instability, alterations in bone remodeling, and a higher prevalence of predisposing conditions among older adults, such as Paget’s disease of bone or prior radiation exposure (11, 13). Notably, the bimodal age distribution observed in this study likely reflects the distinct epidemiological profiles of different histological subtypes of primary bone cancer. Osteosarcoma, the most common subtype, predominantly affects adolescents and young adults during periods of rapid skeletal growth, whereas chondrosarcoma occurs almost exclusively in adults over 40 years of age, and Ewing sarcoma peaks in the second decade of life (3, 6). The absence of histological subtype data in the present study precludes confirmation of this subtype-driven interpretation; however, the age-incidence pattern is highly consistent with the known epidemiology of these entities. Importantly, the persistence of sex differences in older age groups within the Saudi population underscores that male predominance in bone cancer is not confined to adolescence but extends across the lifespan. Together, these findings highlight that, although bone cancer remains a rare malignancy, older adults—particularly males—represent a distinct epidemiological subgroup, reinforcing the need for age- and sex-stratified surveillance and analytic approaches in future national cancer studies (11, 14).

### 4.4 Regional heterogeneity and population-based patterns

The present study demonstrated measurable regional heterogeneity in the incidence of bone cancer across Saudi Arabia, as reflected by significant differences in crude and age-standardized incidence rates between administrative regions. While overall incidence remained low nationwide, selected regions consistently exhibited higher incidence levels compared with others, indicating that bone cancer burden is not uniformly distributed within the population. These findings suggest that regional differences in demographic structure, healthcare access, diagnostic practices, and population density may contribute to observed variability.

Similar regional disparities have been documented in population-based cancer registry studies worldwide, where geographic variation in bone cancer incidence persists even after age standardization. Global cancer surveillance data indicate that differences in incidence across regions are often influenced by variations in population age structure, registry completeness, and diagnostic intensity, particularly for rare malignancies such as bone cancer (15). In addition, regional heterogeneity may reflect differences in the prevalence of underlying risk factors, including hereditary predisposition, prior radiation exposure, and bone-related disorders (16).

However, it is essential to acknowledge that in registry-based studies conducted within developing or transitioning healthcare systems, observed regional differences may be substantially influenced by systematic biases rather than true epidemiological variation. Specifically, regions with limited access to specialized oncology and orthopedic diagnostic centers—such as Jazan, Northern Borders, and Najran—may experience underreporting of bone cancer cases due to diagnostic delays, misclassification, or referral of patients to tertiary centers in larger cities (e.g., Riyadh, Jeddah). Conversely, regions hosting major referral hospitals may exhibit artificially elevated incidence rates if cases diagnosed at these centers are attributed to the hospital’s region rather than the patient’s region of residence. Furthermore, temporal improvements in registry coverage and diagnostic capacity over the 17-year study period may have contributed to apparent increases in incidence that reflect enhanced case ascertainment rather than genuine epidemiological change. These potential sources of bias should be considered when interpreting the regional and temporal patterns described in this study, and future analyses should incorporate registry completeness indicators where available.

Importantly, the statistically significant regional differences observed in this study reinforce the value of subnational analyses when examining rare cancers. National-level averages may obscure meaningful geographic variation, underscoring the need for region-specific surveillance and targeted epidemiological investigation. Such approaches are essential for refining cancer control strategies and improving early detection in regions with persistently elevated incidence patterns (17).

### 4.5 Implications for surveillance, diagnosis, and public health planning

Despite the overall rarity of bone cancer, the present study highlights the importance of robust population-based cancer surveillance for accurately characterizing its epidemiological patterns. The observed age- and sex-specific distributions, together with regional heterogeneity in incidence rates, underscore that rare malignancies can exhibit meaningful population gradients that may be overlooked without long-term, high-quality registry data (18). These findings reinforce the critical role of national cancer registries in supporting evidence-based cancer control strategies and monitoring temporal and geographic variations in disease burden.

From a public health perspective, the identification of distinct high-risk age groups—particularly adolescents and older adults has important implications for diagnostic awareness and timely referral, especially in regions demonstrating persistently higher incidence patterns. Integrating epidemiological evidence into national cancer planning frameworks, strengthening registry completeness, and standardizing diagnostic practices are essential steps toward improving early detection and optimizing resource allocation for rare cancers such as bone malignancies (18, 19).

### 4.6 Comparison with international epidemiology

The epidemiological patterns of bone cancer observed in Saudi Arabia are broadly consistent with those reported in international population-based studies. Similar to global findings, the present study demonstrated a clear male predominance, a characteristic bimodal age distribution with a pronounced adolescent peak, and a secondary increase in incidence among older age groups. These features have been consistently documented across multiple regions worldwide, including Europe, North America, and East Asia, suggesting that the fundamental age- and sex-related patterns of bone cancer are largely preserved across populations (11, 20).

However, despite similarities in overall incidence patterns, international cancer registry data indicate that rare malignancies such as bone cancer often exhibit geographic variability influenced by population structure, registry completeness, and healthcare systems. The marked regional heterogeneity identified within Saudi Arabia therefore aligns with global evidence showing that national averages may mask meaningful subnational differences, reinforcing the importance of detailed, population-based surveillance when comparing cancer epidemiology across countries (11, 20).

### 4.7 Methodological strengths and study limitations

A key strength of the present study lies in its use of nationwide, population-based cancer registry data spanning 17 years, which minimizes selection bias and allows for robust assessment of temporal, age-specific, and regional patterns of bone cancer incidence. The availability of standardized incidence measures, including age-specific rates and age-standardized incidence rates, enhances the comparability of findings across regions and between sexes, providing a comprehensive epidemiological overview of this rare malignancy within the Saudi population (21).

Nevertheless, several limitations should be acknowledged. As with most registry-based studies, the analysis is constrained by the availability and granularity of recorded variables. Information on tumor subtype, stage at diagnosis, treatment modalities, and individual-level risk factors was not available, limiting causal inference and preventing more detailed subtype-specific analyses. In addition, the rarity of bone cancer increases susceptibility to random annual fluctuations, particularly at the regional level, which may influence the stability of incidence estimates despite long-term aggregation (22). These considerations underscore the importance of cautious interpretation and highlight the need for complementary analytic and clinical studies to further elucidate etiological and prognostic determinants of bone cancer. In particular, regions with small populations (e.g., Al-Baha, Najran, Al-Jouf) produce highly variable annual rates owing to low case counts, and observed statistical differences involving these regions should be interpreted with appropriate caution. Furthermore, no formal trend analysis (e.g., joinpoint regression or Poisson time-series models) was applied; consequently, descriptions of temporal changes in incidence should be regarded as observational rather than statistically tested trends. The absence of histological subtype data in the SCR dataset precluded analysis by specific tumor types (e.g., osteosarcoma, chondrosarcoma, Ewing sarcoma). Given that the epidemiology of bone cancer varies substantially by subtype—osteosarcoma peaks in adolescence, while chondrosarcoma predominates in older adults—future registry efforts should incorporate morphology coding to enable subtype-specific analyses.

## 5 Conclusion

This nationwide population-based study shows that, although bone cancer is rare in Saudi Arabia, its incidence varies significantly by age, sex, and geographic region. A consistent male predominance was observed, along with a bimodal age distribution characterized by an adolescent peak and a secondary increase in older age groups.

Importantly, marked regional heterogeneity was identified, with persistently higher incidence in selected regions and consistently lower rates in others, even after age standardization. These findings underscore the importance of long-term, age, sex, and region-stratified cancer surveillance to improve understanding of bone cancer epidemiology and support evidence-based cancer control in Saudi Arabia.

## Data Availability

Publicly available datasets were analyzed in this study. This data can be found here: https://shc.gov.sa/en/NCC/Activities/Pages/NewAR.aspx.
